# Mechanism by which TRAF6 Participates in the Immune Regulation of Autoimmune Diseases and Cancer

**DOI:** 10.1155/2020/4607197

**Published:** 2020-11-26

**Authors:** Jingjing Wang, Xinjie Wu, Mengyu Jiang, Guixiang Tai

**Affiliations:** Department of Immunology, College of Basic Medical Science, Jilin University, Xinjiang Street 125, Changchun 130021, China

## Abstract

Tumor necrosis factor (TNF) receptor-associated factor 6 (TRAF6), an E3 ubiquitin ligase, is a signal transduction molecule shared by the interleukin-1 receptor (IL-1R)/Toll-like receptor (TLR) family and the TNFR superfamily. TRAF6 has a unique TRAF domain and RING finger domain that mediate intracellular signaling events. In the immune system, TRAF6-mediated signaling has been shown to be critical for the development, homeostasis, and activation of a variety of immune cells, including B cells, T cells, dendritic cells, and macrophages. Although the pathogenesis and etiology of autoimmune diseases and cancer are not fully understood, it is worth noting that existing studies have shown that TRAF6 is involved in the pathogenesis and development of a variety of these diseases. Herein, we reviewed the role of TRAF6 in certain immune cells, as well as the function and potential effect of TRAF6 in autoimmune diseases and cancer. Our review indicates that TRAF6 may be a novel target for autoimmune diseases and cancer.

## 1. Introduction

Tumor necrosis factor (TNF) receptor-associated factor 6 (TRAF6), an E3 ubiquitin ligase, is a member of the tumor necrosis factor receptor-associated factor (TNFR) family. TRAF6 is considered to be the only signal transduction-related ligase of the TNFR superfamily and the interleukin-1 receptor (IL-1R)/Toll-like receptor (TLR) superfamily within the TNFR family [1, 2]. TRAF6 can activate multiple signaling pathways, such as NF-*κ*B [3]. TRAF6 also plays indispensable roles in regulating innate and adaptive immunity, embryonic development, tissue homoeostasis, and bone metabolism. TRAF6-deficient mice display various developmental abnormalities during embryogenesis, including osteopetrosis [4], failure of neural tube closure [5], defective formation of skin appendices [6], defects in the lymphoid compartment, and absence of mature thymic epithelial cells [7]. One hundred percent of TRAF6-deficient mice experience death within 15 days after birth. In mammals, TRAF6 is abundantly expressed in the brain, lungs, liver, skeletal muscle, and kidney, and it is also found in the heart, spleen, and testes. In the immune system, TRAF6 has been shown to be critical for the development, homeostasis, and activation of various immune cells, including T cells, B cells, dendritic cells, and macrophages [8].

Autoimmune diseases (ADs) are a series of complex chronic inflammatory diseases characterized by the immune response to self-antigen, and cause damage to tissues [9]. Over 100 ADs that affect 5-10% of the world's population [10] have been identified, including rheumatoid arthritis (RA), systemic lupus erythematosus (SLE), ankylosing spondylitis (AS), inflammatory bowel disease (IBD), Sjögren's syndrome (SS), type 1 diabetes (T1D), autoimmune hepatitis (AIH), multiple sclerosis (MS), and primary Sjögren's syndrome (pSS). So far, the cause of ADs is still unclear. Studies have shown that abnormal immune responses in ADs may be caused by the interaction between genetic and environmental factors [11]. Although the understanding of the etiological mechanisms of autoimmune diseases are constantly evolving and progress in treatment is being made [12–14], the long-term prognosis is unsatisfactory for most patients. Therefore, it is necessary to explore new individualized therapeutic targets for clinical use. The role of TRAF6 in immune cells has been extensively studied over the last two decades [15]. Current researches indicate that TRAF6 could be involved in the pathogenesis of a variety of autoimmune diseases, including RA, SLE, LN, and MS. In addition to autoimmune diseases, accumulating evidence indicates that TRAF6 is associated with the progression of various types of tumors, including breast cancer, hepatocellular carcinoma, and lung cancer. This review summarizes the functional roles of TRAF6 in regulating immunocytes and in the pathogenesis of ADs and cancer as well as the possibility of TRAF6 as a potential promising target for future AD and cancer treatment.

## 2. Structure of TRAF6 and the Ubiquitin-Proteasome System

**Figure 1:**
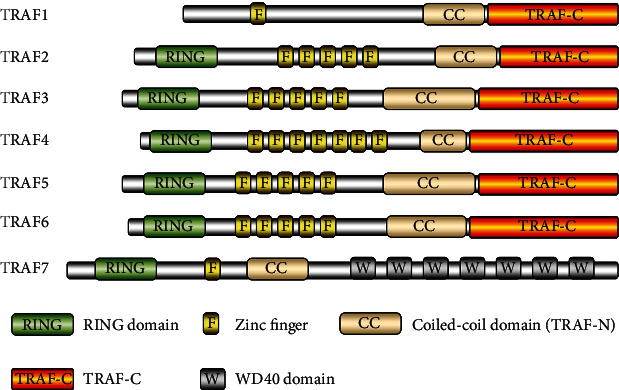
Structure of the TRAF family members. Based on the data by Xie [24]. The TRAF family includes seven family members (from TRAF1 to TRAF7). The proteins contain several domains: a zinc finger, a coiled-coil (TRAF-N), TRAF-C, and a RING.

The TRAF family includes seven members from TRAF1 to TRAF7 (Figure 1). TRAF proteins contain several domains: a zinc finger, a coiled-coil (TRAF-N), TRAF-C, and a RING. The main feature of the TRAF family proteins (except for TRAF1) is the RING homology domain at the N terminus [16], which constitutes the core of E3 ubiquitin ligase activity [17]. The TRAF domain, another feature of the TRAF family (except for TRAF7) at the C terminus, is subdivided into two distinct subdomains: the TRAF-N domain, which is a coiled-coil domain, and the TRAF-C domain, which is composed of seven to eight antiparallel *β*-strand folds. This domain is responsible for protein-protein interactions, including TRAF oligomerization as well as interactions with upstream regulators and downstream effectors [18, 19].

TRAF6 is composed of 522 amino acids with a molecular weight of approximately 60,000 Da. TRAF6 is the only TRAF family member that participates in the signal transduction of both the TNFR superfamily and the IL-1R/TLR superfamily and could regulate adaptive immunity, innate immunity, and bone homeostasis [1]. TRAF6 has the least homology with a prototypical TRAF domain sequence. TRAF2, TRAF3, and TRAF5 bind to the membrane distal domain at the cytoplasmic tail of CD40 and RANK, while TRAF6 interacts with the membrane proximal domain [20]. The differences in the degrees of the directions of the bound peptides have shown that there were marked structural differences between receptor recognition by TRAF6 compared to the other TRAFs [21]. For example, the TRAF-C domain of TRAF6 can interact with RANK and c-Src. As a result, this interaction promotes the activation of c-Src for tyrosine phosphorylation of c-Cbl and Cbl-b, but the phosphorylation is dependent on the TRAF-N domain of TRAF6 [22]. Scavenger receptor A (SRA) inhibits TRAF6 dimerization and ubiquitination by directly interacting with the TRAF-C domain of TRAF6, thereby inhibiting the activation of the TLR4-induced transcription factor NF-*κ*B, causing inflammatory gene expression to be downregulated [23].

**Figure 2:**
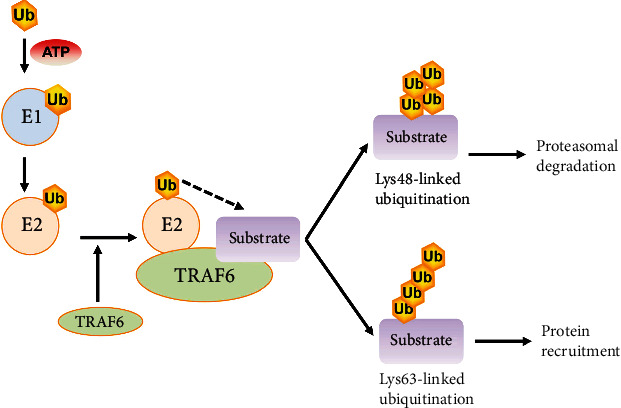
The ubiquitination of TRAF6. Based on the data by Dainichi et al. [28]. Firstly, the E1 enzyme forms a thiol ester bond with a ubiquitin. Next, the activated ubiquitin is transferred to E2. Then, TRAF6 (the E3 enzyme) functions as a scaffold for the binding of both E2 and the target molecule, facilitating the transfer of ubiquitin from E2 to the target protein. Finally, TRAF6 mediates the addition of lysine-linked polyubiquitin chains to the substrates, commonly via K48-linked polyubiquitination or K63-linked polyubiquitination. The K48-linked polyubiquitin causes proteasomal degradation. The K63-linked polyubiquitin recruiting protein is used for further posttranslational modifications.

It has been demonstrated that the ubiquitin-proteasome system consists of 3 primary enzymes: a ubiquitin-activating enzyme (E1), a ubiquitin-conjugating enzyme (E2), and a ubiquitin-protein ligase (E3) [25]. TRAF6 has been confirmed to function as an E3 ubiquitin ligase. TRAF6 is responsible for the interaction with specific substrates, which then act in concert with E1, E2, and ubiquitin to place ubiquitin chains on the substrate (Figure 2). Two main types of ubiquitination have been discovered to regulate intracellular signal transduction [26]. The classic ubiquitination process uses Lys48- (K48-) linked polyubiquitin, which could produce the protein targeted by 26S proteasome for degradation [27]. TRAF6 and the Ubc13-Uev1a E2 complex generate K63-linked polyubiquitin chains, which recruit proteins through their selective binding of a ubiquitin-binding domain (UBD) as a novel posttranslational modification without causing proteasomal degradation [28].

## 3. TRAF6 Signaling Mechanism

**Figure 3:**
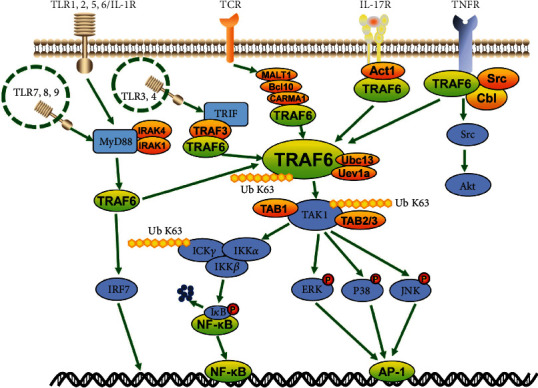
Overall depiction of the TRAF6-mediated signaling pathways. TRAF6 has been identified as a downstream adaptor of multiple receptor families with immunoregulatory functions, including members of the TNFR superfamily, the TLR family, IL-17R, and TCR. These receptors activate TRAF6 by binding directly to TRAF6 or by linking to an intermediate protein. TRAF6 catalyzes the K-63 polyubiquitination of TAK1 by means of Ubc13 and Uev1a. TAK1 forms a complex with TAB1 and TAB2 or TAB3, which phosphorylates and fully activates TAK1. Subsequently, I*κ*B can be phosphorylated and degraded by the activation of the IKK complex, activating NF-*κ*B. However, it can also activate the MAPK pathway, including the ERK pathway, the JNK pathway, and the p38 pathway. In addition, TRAF6 may also directly activate the PI3K and IRF pathways. In this process, TRAF6 conjugates K63-linked polyubiquitin chains (Ub K63) (e.g., IKK*β*, TAK1, IRAK, and TRAF6) and K48-linked polyubiquitin chains (Ub K48) (e.g., I*κ*B) to transduce the signal.

TRAF6, an unconventional E3 ubiquitin ligase, binds to ubiquitin via a thioester bond in a catalytic reaction and transmits ubiquitin to the relative substrate, thereby participating in a series of signal transductions in immune cells and nonimmune cells. The general signaling roles played by TRAF6 in immune cells are summarized here, but some of them are dependent on context or type-specific cells. In addition to the TLR/IL-1 family and the TNFR superfamily, TRAF6 is also involved in the signaling pathways of IL-17R and TCR (Figure 3). TRAF6 is widely expressed in various immune cells, but most immune cells limit TRAF6 activity by selectively expressing receptors for activating it.

TRAF6 interacts directly with some receptors through a binding motif, and links to other receptors through an intermediate protein. It also interacts directly with TNFR superfamily members, primarily RANK and CD40, via the TRAF6 binding motif. The CD40 cytoplasmic tail is associated with the activation of TRAF6 [29]. The binding of RANKL to RANK induces the ternarization of RANK and the recruitment of the adaptor protein TRAF6 through the three TRAF6 binding sites in its C-terminal cytoplasmic tail as described previously [30], thereby initiating the downstream signaling cascade. In other cases, TRAF6 is linked to the receptor via intermediate proteins. The IL-17R family activates TRAF6 via Act1 [31]. TCR activates TRAF6 via CARMA1, Bcl10, and MALT1 [32]. Additionally, some TLR family members (e.g., TLR3/4) are linked by TRIF, recruit TRAF3, and activate TRAF6. Other TLR family members and some IL-1R superfamily members (e.g., TLR1/2/4/5/6, TLR7/8/9, and IL-1/18/33R) initiate MyD88 recruitment, bind to and activate IL-1 receptor-associated kinase 1 or 4 (IRAK), and thereby activate TRAF6 [33–36].

Upon activation, TRAF6 can complex with the ubiquitin-conjugating enzyme Ubc13 and its variant Uev1a, conjugating K63-linked polyubiquitin chains to transduce the signal and attach K63-linked ubiquitin chains to lysine residues on various target proteins, including inhibitory kappa B kinase (IKK*γ*, also known as NEMO), TGF-*β*-activated kinase 1 (TAK1), IRAK1, and TRAF6 itself [37, 38]. TAK1 and TAK1-binding proteins TAB1 and TAB2/3 form the TAB1/TAK1/TAB2/3 complex that activates the IKK*α*/IKK*β*/IKK*γ* complex and phosphorylates I*κ*B, which lead to the degradation of I*κ*B and the activation of the transcription factor NF-*κ*B [39, 40]. Simultaneously, TRAF6 can also activate downstream interferon regulatory factor 7 (IRF7) via the TLR7/8/9-MyD88 pathway [41]. In addition, TAK1 can also lead to the activation of mitogen-activated protein kinase (MAPK) pathways, including the extracellular signal-regulated kinase pathway, the extracellular regulated protein kinase (ERK) pathway, the c-Jun N-terminal kinase (JNK) pathway, and the p38 pathway [42]. These MAPK pathways are involved in the activation of transcription factor AP-1. Moreover, RANK and CD40 could activate protein kinase B (Akt) in a variety of cell types via TRAF6. RANK or CD40 forms a complex with the Cbl family of scaffolding proteins and TRAF6, depending on the Src kinase activity. Additionally, Cbl brings phosphoinositide 3-kinase (PI3K) to the receptor complex and activates Akt [43, 44].

## 4. Regulatory Effects of TRAF6 on Various Immune Cells

TRAF6 is widely expressed in a variety of immune cells, including T cells, B cells, dendritic cells (DCs), and macrophages [8]. Herein, we summarize the immune-related regulatory role of TRAF6 in these cells, and the recent evidence, which amply confirmed the mechanism.

### 4.1. T Cells

To study the specific role of TRAF6 in T cells, TRAF6-*Δ*T mice (with the TRAF6 deletion specific to T cells) were established by crossing floxed TRAF6 mice with CD4-*Cre* transgenic mice [45]. The TRAF6-*Δ*T mice show splenomegaly and lymphadenopathy, with a significant decrease in the percentage of CD8^+^ T cells but an increase in the number of CD4^+^ T cells. The T cell-specific deletion of TRAF6 results in multiorgan inflammatory diseases accompanied by mononuclear cell infiltration, including in the intestine, liver, lungs, and kidneys. Abnormal Th2 cytokine production, such as IL-4 and IL-10, can also be observed in the infiltrated organs. In addition, compared with wild-type T cells, TRAF6-deficient T cells exhibit hyperactivation of the PI3K-Akt pathway, which becomes resistant to suppression by CD4^+^CD25^+^ Treg cells. The extensive role of TRAF6 in T cells has been gradually recognized with the subset-specific mechanism summarized as follows.

#### 4.1.1. CD4^+^ T Cells

After an initial stimulation by an antigen, naive CD4^+^ T cells may differentiate into different T helper (Th) lineages, including Th1, Th2, Th9, Th17, Th22, and Tregs and perform different functions. The direction of differentiations is regulated by various factors such as the nature of the antigen and the hormones and cytokines in the local environment. Various types of cytokines and the balance among cytokines play an important regulatory role in the differentiation of Th cells. A variety of evidence has shown that TRAF6 plays an essential role in the differentiation of CD4^+^ T cells. NLRC3, a non-inflammasome-forming member of the NLR (nucleotide-binding domain and leucine-rich-repeat-containing) innate immune receptor family, mediates TRAF6-dependent CD4^+^ T cell signaling and metabolism. NLRC3 limits IFN-*γ* and TNF expression in CD4^+^ T cells, as well as the proliferation of Th1 and Th17 cells, by reducing TRAF6 ubiquitination to interfere with NF-*κ*B signaling [46]. OX40 (also known as tumor necrosis factor receptor super family 4, *Tnfrsf4*) acts as a potent inducer of Th9 cells in a TRAF6-dependent manner. OX40 ligation together with TGF-*β* and IL-4 convert up to 80% of CD4^+^ T cells into Th9 cells. A mechanism analysis revealed that the OX40 linkage activates TRAF6 and mediates the activation of NF-*κ*B-inducing kinase (NIK). NIK activation results in the processing of p100 to generate the p52 subunit which, together with RelB, forms the transcriptionally active p52-RelB complex. This noncanonical NF-*κ*B pathway is critical for OX40-induced Th9 differentiation [47]. It is well known that the synergy of TGF-*β* and IL-6 is required for the generation of Th17 cells, and IL-2 is a known inhibitor of Th17 differentiation. CD4+ T cells lacking TRAF6 showed a specific increase in Th17 differentiation, which is caused by the increased TGF-*β*-induced Smad2/3 activation and TGF-*β*-dependent IL-2 downregulation. Notably, TRAF6-deficient T cells could produce normal amounts of Foxp3-expressing cells under the conditions of the iTreg differentiation that provided exogenous IL-2 [48]. Additionally, TRAF6-deficient mice showed severe defects in Treg development in the thymus [49]. In Tregs, TRAF6 maintains Foxp3 and suppresses the pathogenic-Th2 type conversion of Tregs. In lymphopenic or inflammatory conditions, TRAF6-/- Tregs tend to lose Foxp3 with these cells transformed into Th2-like IL-4-producing cells. As a result, Treg-specific TRAF6-deficient mice develop various diseases such as allergic skin diseases, arthritis, and lymphadenopathy [50].

#### 4.1.2. CD8^+^ T Cells

CD8^+^ T cells are central players in controlling pathogen infections and cancer. The T cell-specific deletion of TRAF6 shows the unique role of TRAF6 in the differentiation and metabolism of CD8^+^ T cells. TRAF6-deficient CD8^+^ T cells show altered fatty acid metabolism, and consequently, the CD8^+^ T cells have a defective ability to generate long-lived memory cells, which could be restored by the use of the antidiabetic drug metformin [51]. Although naïve TRAF6-*Δ*T CD8^+^ T cells show normal survival when transferred into normal T cell pools, recent studies have shown that naïve TRAF6-*Δ*T CD8^+^ T cells show defective lymphopenia-induced proliferation (LIP) [52]. Specifically, IL-18 potentiates LIP in vivo and synergizes with high doses of IL-7 to induce LIP-like proliferation in vitro. IL-7 and IL-18 act synergistically to upregulate the expression of the IL-18 receptor (IL-18R) genes, thereby enhancing IL-18 activity. In this case, IL-18R signaling increases the TRAF6-dependent activation of PI3K/Akt activation and makes naïve CD8^+^ T cells sensitive to noncognate self-peptide ligands. Consequently, the TRAF6-mediated pathway is required for both in vivo LIP and in vitro IL-7/IL-18-mediated synergy. This evidence suggests that there are multiple TRAF6-dependent signaling pathways in T cells. Different pathways may play different roles in T cell proliferation, differentiation, metabolism, and homeostasis.

### 4.2. B Cells

Some studies have shown that TRAF6 is involved in B cell development, maturation, homeostasis, and function. By blocking the binding of CD40 to TRAF6 in B cells, it has been found that the binding of TRAF6 to CD40 was able to induce IL-6 and antibody secretion. TRAF6 mediates its secretion of CD40-stimulated antibodies mainly through its effect on IL-6 production by B cells. The combination of TRAF6 and CD40 upregulates B7-1, but other surface molecules do not. Surprisingly, the activation of NF-*κ*B and c-Jun kinase in CD40-mediated B cells was not affected by the disruption of CD40-TRAF6 binding [53]. Subsequently, it has been discovered that the consensus binding sites of TRAF2 and TRAF3 account for the majority of signaling via CD40, but the TRAF6 binding site appears to be redundant in the activation of the p38 and NF-*κ*B signaling pathways [54]. However, the generation of high-affinity antibodies and long-lived bone marrow plasma cells require the recruitment of TRAF6 to the CD40 cytoplasmic domain [55]. TRAF6 resides not only in the cellular cytoplasm but also in the nucleus of normal and malignant B lymphocytes. Nuclear TRAF6 is modified by small ubiquitin-related modifier-1, then interacts with histone deacetylase 1, and eventually represses c-Myb-mediated transactivation. Therefore, nuclear TRAF6, as a negative regulator, may maintain cellular signal homeostasis by inhibiting transcriptional regulators [56]. B cell-specific TRAF6-deficient mice showed a significant reduction in the number of mature B cells in the bone marrow and spleen. Furthermore, the TRAF6-deficient B cell progenitors are unable to produce CD5^+^ B-1 cells. B cell-specific TRAF6-deficient mice are severely impaired in both T cell-dependent antigenic responses and T cell-independent antigenic responses, possibly due to the complete absence of CD5^+^ B-1 cells in the peritoneal cavity. This indicates that TRAF6 is required for CD5^+^ B-1 cell development and the maintenance of a mature B cell bank [57].

Activation of NF-*κ*B by latent membrane protein 1 (LMP1) of the Epstein-Barr virus is critical for the survival of Epstein-Barr virus-infected B lymphocytes. LMP1 is also involved in the progression of autoimmune diseases such as systemic lupus erythematosus. By detecting the LMP1-induced levels of NF-*κ*B activation in a variety of gene-specific knockout mouse embryonic fibroblasts, it was found that LMP1 activation of NF-*κ*B can be independent of TRAF2 and TRAF5, but is dependent on TRAF6 or IRAK1 [58]. Further research indicates that two regions of the cytoplasmic carboxyl tails of LMP1, the C-terminal-activating regions 1 and 2 (CTAR1 and CTAR2), are responsible for NF-*κ*B activation. LMP1 activates NF-*κ*B mainly through the classical CTAR2/TRAF6/TAK1/IKK*β* pathway, in addition to the noncanonical CTAR1/TRAF3/NIK/IKK*α* pathway [59]. Using TRAF6-deficient B cells, it was determined that TRAF6 is required for LMP1-mediated B cell activation and associated with the CTAR1 subdomain of LMP. LMP1, as a constitutively active functional mimetic of CD40, mediates TRAF6-dependent signaling that requires the TRAF6 receptor binding domain, but the CD40-mediated signaling pathway does not require the presence of this domain [60]. Similarly, in a B cell-specific TRAF6-deficient mouse model, TRAF6 is an important mediator of LMP1 B cell function in vivo. LMP1 is more dependent on TRAF6 than CD40 to promote the B cell effector function [61]. In short, it would be beneficial to target TRAF6 to disrupt downstream LMP1 signaling. Since LMP1 does not have a canonical TRAF6 binding site, it is also possible to disrupt the association of TRAF6 and LMP1 while maintaining its binding to CD40.

### 4.3. Dendritic Cells

DCs, as a kind of antigen-presenting cell (APC), could activate the innate and adaptive immune responses after infection occurs and maintain immune tolerance. TRAF6 not only plays a key role in DC maturation, including the expression of the surface markers, secretion of cytokines, and the ability to stimulate naïve T cells, but is also essential for the development of DC subsets.

#### 4.3.1. TRAF6 Contributes to the Maturation of DCs

**Figure 4:**
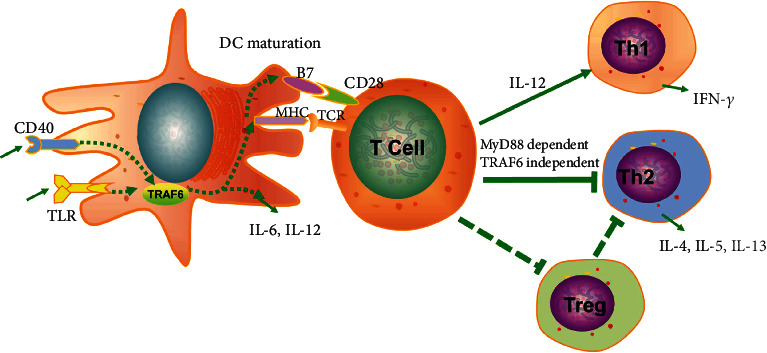
The role of TRAF6 in dendritic cells. TRAF6 is a key regulator of TLR, CD40, and RANK, which regulates DC maturation and interaction with T cells. TRAF6 regulates DC maturation by upregulating the expression of B7 and MHC molecules and secreting the cytokines IL-12 and IL-6. The activated DCs interact with T cells, causing CD4^+^ T cells to polarize to Th1. DCs in TRAF6-deficient mice are unable to upregulate MHC and B7.2 and secrete IL-12 in response to stimulation with CD40L or various TLR ligands. Moreover, the ability of TRAF6-deficient DCs to activate antigen-specific CD4^+^ T cells to become IFN-*γ*-producing Th1 cells is insufficient. However, they have no effect on the development of Th2 cells. In addition, TRAF6 directs DCs to maintain intestinal and lung immune tolerance by balancing the induction of Tregs and Th2 cellular immunity.

TRAF6-deficient DCs can directly exhibit the role of TRAF6 to some extent. TLR ligands or CD40L induces the upregulation of surface molecules such as MHC class II and B7.2 on wild-type DCs in vitro, and upregulation is severely impaired in TRAF6-deficient DCs. Similarly, the administration of LPS or the anti-CD40 antibody in vivo induces DC maturation in wild-type mice but not in TRAF6 knockout mice [62]. These results imply that TRAF6 is required for DC maturation in response to microbial components, such as LPS or CD40L, both in vitro and in vivo. Moreover, while wild-type DCs activated by various TLR ligands or CD40L produce a substantial amount of the inflammatory cytokines IL-6 and IL-12, DCs generated from TRAF6 knockout mice exhibit a severe cytokine production defect [62]. Therefore, CD40L and TLR ligands stimulate cytokine production from DCs in a TRAF6-dependent manner (Figure 4).

Another hallmark of DC maturation is their ability to stimulate T cell activation. Restimulated T cells primed in vivo with LPS-treated wild-type splenic DCs show remarkably increased IFN-*γ* production compared to that of T cells primed with untreated wild-type splenic DCs. In contrast, restimulated T cells primed with either LPS-treated or untreated TRAF6-/- splenic DCs exhibit a low amount of IFN-*γ* production [62]. These results indicate that the splenic DCs in TRAF6-*Δ*DC mice exhibit deficiencies in driving acute Th1 immunity. Furthermore, TRAF6-/- DCs do not prevent TLR from inducing Th2 cell development, but MyD88-/- DCs suppress Th2 development. This suggests that the TLR-initiated signaling that is critical for the generation of the negative signal for Th2 cell development occurs via an MyD88-dependent, TRAF6-independent pathway [63] (Figure 4). Similarly, an analysis of small intestinal lamina propria tissue gene expression from TRAF6 *-Δ*DC and littermate control mice older than 8 weeks revealed that the Th1 cell-associated factor IL-12 and IFN-*γ* decrease significantly, while the Th2 cell-associated factors IL-13, IL-5, and IL-4 increase significantly [64] (Figure 4). Furthermore, TRAF6-*Δ*DC mice specifically exhibit Th2-related inflammation and fibrosis in the small intestine. This disease phenotype is associated with Treg homeostasis. This disease can be ameliorated by broad-spectrum antibiotic therapy, which initially indicates that TRAF6-*Δ*DC associated inflammation is triggered by the intestinal commensal microbiota. Notably, MyD88-*Δ*DC mice have no similar performance in the small intestine, suggesting that upstream of the disease is TLR independent. As with the intestine, the absence of TRAF6 expression by DCs led to the spontaneous generation of the Th2-associated immune responses in the lungs [65]. The relative mRNA levels of the Th1-associated cytokine IL-12*β* were reduced in TRAF6-∆DC lungs compared to control lungs, and the mRNA level of profibrogenic factor Igf-1 and the Th2-associated cytokines IL-13 and IL-5 also significantly increased in TRAF6-∆DC lungs. TRAF6-dependent signaling is essential to establish and/or maintain immune tolerance in the lungs and small intestine to prevent spontaneous Th2-associated hypersensitivity.

The TRAF6 signaling pathway is not the only pathway associated with DC maturation. Cytokine LIGHT is a type II transmembrane protein belonging to the TNF family that plays a role in inducing the maturation of dendritic cells. Interestingly, LIGHT-mediated DC maturation does not require TRAF6, since the knockdown of TRAF6 does not affect the LIGHT-induced upregulation of CD86 expression [66]. Therefore, other TRAF6-independent signaling pathways still exist in the maturation of DCs.

#### 4.3.2. TRAF6 Is Essential for the Development of DC Subsets

For nearly two decades, TRAF6 has been shown to be essential for the development of multiple tissues and organs. Depending on the differential expression of the CD4^+^CD8*α* molecules, namely CD4^+^CD8*α*^−^, CD4^−^CD8*α*^+^, or CD4^−^CD8*α*^−^ [67], DCs in the mouse spleen can be assigned to one of three distinct subgroups. Among the three DC subpopulations, CD4^+^ DCs predominate in the wild-type spleen, while the CD4^+^ DC population is almost completely absent in the TRAF6 knockout spleen. This phenotype is similar to knockout mice lacking the NF-*κ*B subunit RelB, which is located downstream of TRAF6 activation, suggesting that the TRAF6-RelB signaling pathway may control the development of the CD4^+^ DC subpopulation in the spleen [62]. DCs in the adult thymus are mostly CD8*α*^+^CD11b^−^, but CD8*α*^−^CD11b^+^ DCs were abundantly present in the fetal thymus and they possessed antigen-presenting activity. Thymic CD11b^+^ DCs are dramatically reduced in TRAF6-deficient fetal thymus compared with the wild type. These results indicate that TRAF6 activation is required for the development of CD11b^+^ DCs in the fetal thymus [68].

### 4.4. Macrophages

Macrophages have been confirmed to participate in tissue development and inflammation in response to pathogenics, cancer, and organ transplantation. Accumulating evidence has identified the role of TRAF6 in macrophages in recent years on a large scale. For example, Mason et al. report that, compared to the wild-type, TRAF6-deficient mice fail to produce IL-12 when stimulated by the *Toxoplasmagondii* antigen (STAg). TRAF6-deficient macrophages are also unable to secrete IL-12 in response to STAg. Therefore, TRAF6-dependent activation is required for the production of IL-12 in macrophages in response to STAg [69]. Mannose-terminated lip arabinose (Man-LAMs) from *M. tuberculosis* inhibits LPS-induced IL-12 production in murine RAW264.7 macrophages by inhibiting IRAK-TRAF6 interaction, I*κ*B-*α* phosphorylation, and the nuclear translocation of c-Rel and p50 [70]. Similarly, HLA-B-associated transcript 3 (BAT3), which is also known as Scythe or Bag6, inhibits the homooligomerization of TRAF6 as well as the interaction between TRAF6 and TAK1, thereby functioning as the negative regulator of LPS-induced macrophage activation. The inhibited TRAF6-mediated signaling by BAT3 may be related to the direct binding of BAT3 to TRAF6 and the subsequent blocked interaction with downstream signaling molecules, or it may be due to the presence of a third-factor-mediated BAT3-dependent inhibition of TRAF6 [71]. In addition, major vault protein (MVP), a major component of nuclear ribonucleoproteins, acts as an inhibitor of NF-*κ*B signaling in macrophages. The worsening metabolic disorders caused by MVP deficiency are accompanied by an increase in macrophage infiltration and an increase in the inflammatory response in the microenvironment. In vitro studies have shown that MVP interacts with TRAF6, preventing its recruitment to IRAK1 and subsequent oligomerization and ubiquitination. The overexpression of MVP inhibits the activity of TRAF6 and macrophage inflammation [72].

The fact that Ubc13 is depleted in STAT3-deficient macrophages explains why STAT3 could inhibit the production of proinflammatory cytokines mediated by NF-*κ*B signaling in innate immune cells. Specifically, STAT3 inhibits Ubc13 expression by direct transcriptional repression, thereby inhibiting TRAF6 K63-linked ubiquitination, NF-*κ*B signaling, and RANKL- and LPS-responsive gene expression [73]. The abnormal cleavage of retinoic acid X receptor-alpha (RXR*α*) in tumor cells and tissues produces a truncated RXR*α* (tRXR*α*) with a tumorigenic effect, mainly due to its expression in bone marrow cells leading to IL-6 induction and STAT3 activation. Studies have revealed the broad interaction of tRXR*α* with TRAF6 in the cytoplasm of macrophages, leading to ubiquitination of TRAF6 and subsequent activation of the NF-*κ*B inflammatory pathway [74]. STAT1 is also directly recruited into the TLR signaling pathway through interaction with TRAF6, leading to the activation of STAT1 residue 727 (S727) via phosphorylation, which mediates specific proinflammatory cytokine responses following TLR stimulation. Notably, compared to wild-type macrophages, macrophages produced by mice that replaced the S727 residue with alanine (STAT1 S727A mice) show a significant reduction in TNF-*α* protein production, but no decrease in IL-6 or the RANTES protein [75]. These studies demonstrate an increased incidence of crosstalk between the TLR/IL-1 and JAK/STAT signaling pathways, suggesting that differential activation of STAT family members may contribute to the TLR proinflammatory responses following pathogen challenge.

## 5. The Role of TRAF6 in Autoimmune Diseases

### 5.1. Rheumatoid Arthritis

Rheumatoid arthritis (RA) is a systemic autoimmune disease characterized by the expansion of synoviocytes and inflammatory immune cells that affects approximately 1-2% of the world's population [76]. Although the etiology of RA is unclear, it is considered to occur in the presence of genetic predispositions and environmental factors [77, 78]. In recent years, the role of TRAF6 has been recognized in the pathogenesis of RA.

**Figure 5:**
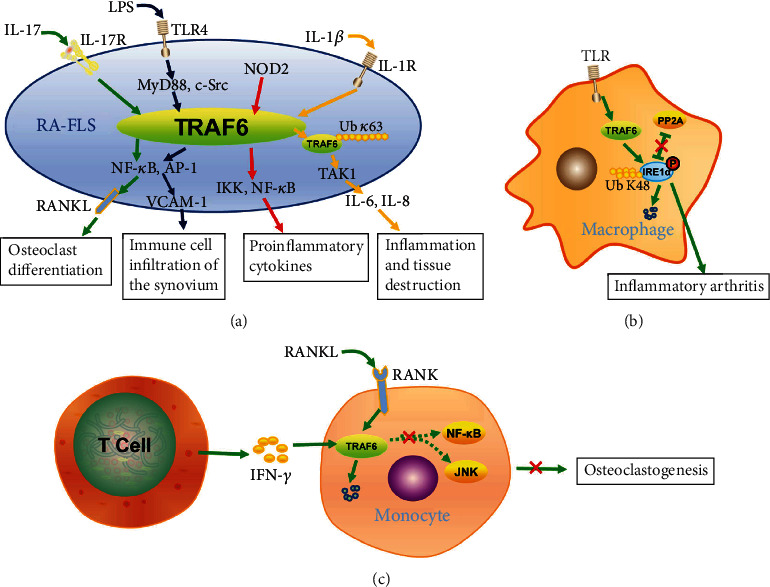
An overview of TRAF6 in the pathogenesis of RA. (a) The role of TRAF6 in RA-FLS on the pathogenesis of RA. LPS regulates NF-*κ*B and AP-1 activation by inducing TLR4/MyD88/TRAF6/c-Src, leading to the induced high expression of VCAM-1 and immune cell infiltration of the synovium. NOD2 protein activates TRAF6, IKK, and NF-*κ*B, leading to the secretion of inflammatory cytokines. In IL-1*β*-stimulated RA-FLS, the increased autoubiquitination of K63-linked TRAF6 enhances the binding of TRAF6 and TAK1 and induces IL-6 and IL-8 synthesis. IL-17 induces the upregulation of RANKL and osteoclast differentiation mediated by the Act1/TRAF6/NF-*κ*B and AP-1 pathways. (b) IRE1*α* contributes to the development of inflammatory arthritis. In the early phase of TLR stimulation, the TRAF6-mediated K48-linked polyubiquitin chain leads to the degradation of the IRE1*α* protein, thereby inhibiting PP2A recruitment, phosphorylating the IRE1*α* protein, and positively regulating IRE1*α* activation. In the late phase of stimulation, TRAF6 destroys the IRE1*α* protein to terminate signal transduction. (c) IFN-*γ* production by T cell accelerates TRAF6 proteolysis, resulting in the strong inhibition of RANKL-induced NF-*κ*B and JNK activation, and finally inhibiting osteoclastogenesis.

The increased expression of TRAF6 in RA fibroblast-like synoviocytes (RA-FLS) is significantly associated with the severity of synovitis and the number of infiltrating inflammatory cells. Compared with osteoarthritis, multiple molecules involved in TLR signaling are upregulated in the FLS cells of RA patients, including TRAF6 [79]. The role of TRAF6 in RA-FLS contributes to the development of RA (Figure 5(a)). Infection with Gram-negative bacteria has been considered to be an initiator of rheumatoid arthritis. In RA-FLS, LPS regulates NF-*κ*B and AP-1 activation by inducing the formation of the TLR4/MyD88/TRAF6/c-Src complex, leading to the induced high expression of vascular cell adhesion molecule-1 (VCAM-1) and immune cell infiltration of the synovium [80]. The expression levels of the NOD2 gene and protein were found to be significantly increased in RA-FLS. When the NOD2 gene expression was inhibited, the levels of TRAF6, IKK, and NF-*κ*B were also decreased [81]. In IL-1*β*-stimulated human RA-FLS, the TRAF6 expression remained unchanged, but the K63-linked autoubiquitination of TRAF6 increased. When inhibiting the K63-mediated autoubiquitination, the binding of TRAF6 and TAK1 is inhibited, which abrogates IL-1*β*-induced IL-6 and IL-8 synthesis in RA-FLS. The autoubiquitination, but not expression, of TRAF6 is critical in IL-1*β*-induced RA signaling mechanisms [82]. Th17 cytokines, particularly IL-17, induce the upregulation of RANKL and osteoclast differentiation mediated by the Act1/TRAF6/NF-*κ*B and AP-1 pathways [83]. Blocking the IL-17 and RANKL axis may be a potential new therapeutic target for bone destruction in RA. Additionally, neddylation, a process similar to ubiquitination, is critical in various ADs. In FLSs, TRAF6 neddylation might mediate RA responses by regulating NF-*κ*B activation [84]. Targeting neddylation activation may be a potential therapeutic strategy for the treatment of RA.

The abnormal expression of TRAF6 in a variety of immune cells is associated with the pathogenesis of RA. In RA, macrophages are major sources of inflammatory mediators. The synovial TRAF6 expression level in RA positively correlated with synovitis severity and macrophage density [85]. Macrophages produce inflammatory cytokines by TLR-mediated signaling during RA. Inositol-requiring enzyme 1*α* (IRE1*α*) activation is significantly increased in the macrophages from the synovial fluid of RA patients (Figure 5(b)). Additionally, mice with bone myeloid loss due to the IRE1*α* gene are also protected from inflammatory arthritis. Protein phosphatase 2A (PP2A) is a phosphatase that inhibits IRE1*α* phosphorylation. Further analysis reveals that at the early phase of TLR stimulation, TRAF6-mediated polyubiquitination appears to positively regulate IRE1*α* activation by suppressing PP2A recruitment and IRE1*α* protein degradation. In the late phase of stimulation, TRAF6 destroys the IRE1*α* protein to terminate signal transduction [86]. Activated T cells promote osteoclastogenesis and regulate bone loss in RA through RANKL expression [87]. However, T cell production by IFN-*γ* strongly inhibits osteoclastogenesis by interfering with the RANKL-RANK signaling pathway (Figure 5(c)). This is because IFN-*γ* accelerates TRAF6 proteolysis by participating in the ubiquitin-proteasome pathway, resulting in the strong inhibition of the activation of RANKL-induced transcription factor NF-*κ*B and JNK [15]. The proteins of the RANK/RANKL pathway expressed by neutrophils mediate important functions of neutrophils during the abnormal immune response and bone remodeling in RA, including RANKL, OPG, and RANK. Unexpectedly, healthy and inflammatory neutrophils continue to express TRAF6, which is not a limiting factor for RANK-mediated neutrophil function [88]. In addition, the expression of miR-146a is increased in the peripheral blood mononuclear cells (PBMCs) of RA patients, but the two targets of miR-146a, TRAF6 and IRAK1, express similar levels between RA patients and control individuals [89]. However, the RNA level of miR-146a was decreased in RA tissues [90]. Recently, new studies have found that iguratimod treatment increases miR-146a while reducing cell proliferation and IRAK1 and TRAF6/JNK1 pathways in RA-FLS in a dose-dependent manner. Iguratimod can improve the progression of RA by regulating the expression of IRAK1 mediated by the miR-146a and TRAF6/JNK1 pathway [91]. In addition to miR-146a, miR-146b can promote the expression of TRAF6 and activate NF-*κ*B signaling [92]. miR-345-3p suppressed the activation of the TAK1/p38/NF-*κ*B pathway by targeting TRAF6, and the overexpression of TRAF6 eliminates the biological function of miR-345-3p [93]. miR-125b-5p directly binds to the 3′UTR of TRAF6 mRNA in human osteoarthritic (OA) chondrocytes, and negatively regulates the TRAF6/MAPKs/NF-*κ*B pathway [94]. These findings indicate that targeting miR-146a, miR-146b, miR-345-3p, and miR-125b-5p can be potential treatment strategies for RA. More importantly, TRAF6 can be a potential promising target for RA treatment.

Taken together, TRAF6 is a critical mediator in the inflammatory pathway and activity of osteoclasts. We believe that the pathologic role of TRAF6 in RA is not limited to a certain type of immune cell. Rather, the overall RA pathology can be demonstrated to result from the combined action of TRAF6 on multiple cell types including macrophages, Th17 cells, and synoviocytes.

### 5.2. Lupus Nephritis

Lupus nephritis (LN) is a major complication of systemic lupus erythematosus (SLE). Some studies have shown that decreased miR-146a could upregulate TRAF6 and further contribute to the pathogenesis of LN. TRAF6 is a direct target of miR-146a in kidney tissue. miR-146a rs2910164 interfered with the production of mature miR-146a, thereby promoting the expression of TRAF6 [95]. The expression of miR-146a is significantly decreased in the renal tissues of patients with LN, while the expression of TRAF6 is elevated. The upregulation of miR-146a or downregulation of TRAF6 can significantly inhibit the NF-*κ*B transcriptional activity of glomerular mesangial cells and inhibit inflammatory factor synthesis, such as IL-1*β*, IL-6, IL-8, and TNF-*α*, as well as reduce the chemotactic effects of phagocytes [96]. In addition, compared with healthy controls, LN patients have decreased miR-146a in PBMC, while TRAF6 expression increased. TRAF6 is positively correlated with serum IL-1*β*, IL-6, IL-8, and TNF-*α* activities. miR-146a reduction and TRAF6 upregulation increase the likelihood of the progression and recurrence of end-stage renal disease (ESRD) within one year [97]. Interestingly, miR-146a is enriched in urinary exosomes in patients with LN [98]. Because urine is a potential liquid biopsy medium for patients, the risk of LN is associated with miR-146a, which may be a biomarker and therapeutic target for predicting LN in SLE patients. In addition, circulating miR-203 expression was downregulated in active LN patients. The overexpression of miR-203 inhibited the activation of IL-*β*, IL-6, and TNF-*α* induced by TRAF6 in human mesangial cells (HRMC) and the human renal tubular epithelial cell line (HK-2) [99]. A reduction in circulating miR-203 is a candidate diagnostic biomarker for active LN in humans. Nuclear enriched abundant transcript 1 (NEAT1), a novel lncRNA, accelerated renal mesangial cell injury by directly targeting miR-146b, promoting the expression of TRAF6, and activating the NF-*κ*B signaling in lupus nephritis [92]. This indicates the role of the NEAT1/miR-146b/TRAF6 axis in the pathogenesis of LN. miR-146b may also be a potential therapeutic target for LN.

In addition to the regulatory mechanism of miR-146a on TRAF6, the TLR signaling mechanism is involved in the pathogenesis of LN. (E)-2-(2-Chlorostyryl)-3,5,6-trimethylpyrazine (CSTMP) is a useful agent for the treatment of LN due to the inhibition of the TLR4/MyD88/TAK1/TRAF6/IRAK1 pathway [100]. TANK is a negative regulator of TLR signaling. In TANK-/- macrophages, the TLR-induced polyubiquitination of TRAF6 is upregulated; meanwhile, classical NF-*κ*B activation is also elevated. In addition, TANK-/- mice spontaneously develop lupus autoimmune nephritis. TANK inhibits TLR signaling by controlling TRAF6 ubiquitination, which is involved in the development of LN [101].

### 5.3. Multiple Sclerosis

Multiple sclerosis (MS) is a common inflammatory autoimmune disease that affects the function of the central nervous system (CNS), for which the most common experimental model is experimental autoimmune encephalomyelitis (EAE). Nlrc3-/- mice exhibit worsening EAE, because NLRC3 reduces the K63-linked TRAF6 ubiquitination in CD4^+^ T cells to limit NF-*κ*B activation [46]. The binding of TRAF3 to IL-17R interferes with the formation of the receptor signaling activation complex IL-17R-Act1-TRAF6. This complex significantly inhibits the IL-17-induced expression of inflammatory cytokines and chemokine genes. In contrast, the disruption of this complex leads to the development of EAE [102]. Histone 3 Lys27 trimethyl transferase Ezh2 promotes the ubiquitination of Lys48-linked and the degradation of TRAF6. The deletion of Ezh2 reduces the activation of macrophages and microglia, attenuating the autoimmune inflammation of experimental autoimmune encephalomyelitis [103]. Additionally, both MHCII-CD40-TRAF2/3/5-/- and MHCII-CD40-TRAF6-/- mice (CD40-deficient mice expressing a chimeric CD40 transgene with mutations at the TRAF2/3/5 or TRAF6 binding site, respectively, under the control of the MHCII promotor) show a reduction in clinical signs of EAE and prevent demyelination. However, only MHCII-CD40-TRAF6-/-mice show a decrease in myeloid and lymphoid cell infiltration into the CNS, accompanied by a decrease in TNF-*α*, IL-6, and IFN-*γ* levels [104]. Therefore, blocking the CD40-TRAF6 interaction may be a promising treatment for MS.

### 5.4. Other Autoimmune Diseases

The pathogenesis of various autoimmune diseases is related to the regulation of TRAF6 by miR-146a. The pathogenesis of Graves' orbitopathy (GO) is associated with the complex regulation of miR-146a and TRAF6. The expression level of miR-146a in GO eyelid adipose tissue is significantly higher than that in non-GO tissues [105]. The relative expression of miR-146a in the plasma of GO patients is significantly increased. The overexpression of miR-146a significantly promotes the viability and mitosis of orbital fibroblasts [106]. miR-146a inhibits orbital fibrosis and reduces the expression of TRAF6 by inhibiting the TGF-*β* signaling pathway in orbital fibroblasts [105]. However, the circulating miR-146a of GO patients is reduced [107]. This opposite result still needs further clarification. Silencing miR-146a in B cells effectively ameliorates the clinical myasthenic symptoms in mice with ongoing experimental autoimmune myasthenia gravis. Interestingly, miR-146a inhibition has no effect on the expression of TRAF6 and IRAK1 in B cells. This result indicates that the function of miR-146a in B cells does not involve TRAF6 and IRAK1 [108]. In addition, the miR-146a and TRAF6 genes are significantly overexpressed in the PMBCs of patients with Sjögren's syndrome, while the expression of the IRAK1 gene is significantly reduced. Since IRAK1 is considered to be a key gene in the pathogenesis of systemic lupus erythematosus, the pathogenesis of the two diseases is different [109]. TRAF6 may be a specific biomarker for Sjögren's syndrome.

In addition, the conditional deletion of TRAF6 expression in murine thymic epithelial cells (TRAF6-*Δ*TEC mice) results in autoimmune hepatitis (AIH) [110]. In TRAF6-*Δ*T mice, the loss of TRAF6 is closely related to autoimmune sialadenitis via the activation of the inflammation-induced chemokine-chemokine receptor system, such as Th1 cell attractant chemokine CCL2 and its cognate receptor CCR2 or Th17 cell attractant chemokine CCL20 and its cognate receptor CCR6. Therefore, TRAF6-*Δ*T mice can be an effective model of autoimmune sialadenitis [111]. In macrophages, IRAK1 interacts with TRAF6, which promotes the pathogenesis of SLE by enhancing NF-*κ*B signaling and proinflammatory cytokine production. Inhibition of the activity of the IRAK1-TRAF6-NF-*κ*B pathway ameliorates SLE by attenuating macrophage activity and reducing the overproduction of macrophage inflammatory factors such as IL-1*β*, IL-6, and TNF-*α* [112]. B cells are considered to be a key component leading to the complex pathophysiology of SLE. TRAF6 is significantly overexpressed in the peripheral B cells of SLE patients, and TRAF6 can be used as a biomarker for estimating disease in peripheral B cells [113]. The B cell scaffold protein with ankyrin repeats (BANK1) has been genetically associated with SLE and other autoimmune diseases. Georg et al. found that in mouse splenic B cells, BANK1 colocalizes with TLR7 and TLR9 and has five functional TRAF6 binding motifs that interact with TRAF6. The C-terminal domain of BANK1-full-length and the N-terminal domain of the BANK1-Delta2 are necessary for this binding [114]. These findings suggest that the different binding ability of BANK1 variants to TRAF6 is related to the risk of developing SLE. A chimeric protein vaccine composed of the cholera toxin B subunit fused to proinsulin (CTB-INS) is shown to suppress type 1 diabetes (T1D) onset in NOD mice and upregulate the biosynthesis of the tryptophan catabolic enzyme indoleamine 2,3-dioxygenase (IDO1) in DCs. When TRAF2, TRAF3, and TRAF6 blocking peptides are added to vaccinated DCs, IDO1 biosynthesis is inhibited. The CTB-INS vaccine uses a TNFR-dependent signaling pathway resulting in the suppression of dendritic cell-mediated T1D autoimmunity [115].

### 5.5. TRAF6 as a Therapeutic Target of ADs

Here, we summarize the important role of TRAF6 in immunity and autoimmune diseases. Given the critical role of TRAF6 in DC activation and survival, Treg cell production, and Th17 differentiation, TRAF6 is a suitable therapeutic target for autoimmune diseases. In fact, there have been many attempts to treat autoimmune diseases with TRAF6 as a target. The transfection of lentiviral-TRAF6-shRNA inhibits the expression of TRAF6 in RA-FLS, resulting in decreased proinflammatory cytokines and MMPs such as IL-1*β*, IL-8, IL-6, TNF-*α*, MMP-13, and MMP-3 [116]. In vivo, the inhibition of TRAF6 using mice-specific TRAF6 siRNA inhibit serum anti-collagen II antibodies, MMP-1, MMP-3, and MMP-9, thereby reducing the severity of arthritis and joint inflammation. In vitro, the level of MMP produced by IL-l*β*-stimulated human RA-FLS is reduced by anti-TRAF6 monoclonal antibodies, significantly inhibiting the migration and invasion of RA-FLS [117]. After TNF inhibitor (TNFi) treatment, osteoclast differentiation and activity as well as TRAF6 expression are decreased in RA patients by directly reducing the number of osteoclast precursors and inhibiting the intracellular signaling pathway of TRAF6 [118]. This evidence suggests that reducing the expression of TRAF6 may be a useful strategy for the treatment of autoimmune diseases.

Blocking the interaction of TRAF6 with its upstream or downstream molecules is also an effective treatment strategy for autoimmune diseases. Epigallocatechin-3-gallate (EGCG), a potent anti-inflammatory molecule, inhibits TRAF6-associated K63 autoubiquitination to block the binding of TRAF6 and TAK1 and reduces the polyubiquitination of TAK1 phosphorylation and K48 linkages, thereby inhibiting RA symptoms in rats [82]. In preclinical in vivo mouse models, C25-140, an inhibitor of the TRAF6-Ubc13 interaction, can reduce NF-*κ*B activation, improving the disease outcomes for autoimmune psoriasis and rheumatoid arthritis [119]. The CD40-TRAF6-interacting small molecule inhibitor (SMI) not only reduces the transendothelial migration capacity of monocytes but also reduces central nervous system- (CNS-) infiltrating monocyte-derived macrophages during neuroinflammation. In addition, SMI reduces the severity of EAE symptoms in rats but not in mice. This suggests that the inhibition of the CD40-TRAF6 pathway may represent a beneficial therapeutic strategy to reduce monocyte recruitment and macrophage activation in the CNS, but may not be sufficient to completely prevent the clinical symptoms of EAE. SMI has the potential to act as a combination therapy against MS [120].

## 6. The Role of TRAF6 in Cancer

Multiple studies have found that TRAF6 expression is increased in a variety of tumor tissues compared to normal tissues. A total of 193 cancers (59.6%) are identified to have high TRAF6 expression [121], including uterine fibroids [122], glioma [123], pancreatic cancer [124], breast cancer [125], glioblastoma multiforme (GBM) [126], colorectal cancer [127], and squamous cell carcinoma of head and neck (SCCHN) [128].

TRAF6 is related to the proliferation, invasion, migration, and apoptosis of tumor cells. When it is overexpressed, it can endow cells with at least three of the known hallmarks of cancer, including maintaining proliferation signals, resisting cell death, and inducing angiogenesis. Fifty-three of 90 gastric cancers (58.9%) were found to overexpress TRAF6 [129]. The inhibition of TRAF6 in BGC-823 gastric cancer cells, multiple myeloma cells, and human glioma cell lines can reduce proliferation, invasion, and migration and promote apoptosis, while the overexpression of TRAF6 shows the opposite effect [123, 129, 130]. Similarly, the inhibition of TRAF6 can inhibit the migration and invasion of human lung cancer SPC-A1 cells, MCF-7 breast cancer cell lines, SCCHN cells, and GBM cells and promote their apoptosis [125, 126, 128, 131]. In addition, the downregulation of TRAF6 can destroy the tumorigenicity of pancreatic cancer cells in vitro and in vivo [124].

High expression of TRAF6 is associated with poor prognosis in cancer patients. Han et al. found that the expression of TRAF6 is significantly positively correlated with late N, the pathological stage, and a poor prognosis [129]. High TRAF6 expression leads to poor prognosis in patients with head and neck squamous cell carcinoma and promotes lymphatic metastasis [128]. The survival rate of colorectal cancer patients with high expression of TRAF6 is poor [127]. Moreover, TRAF6 has been identified as an independent prognostic factor for GBM [126]. In addition, cancer cachexia is considered as a side effect of cancer. Bilir et al. first showed that patients with cancer cachexia have elevated levels of TRAF6, which is closely related to overall survival [132].

The abnormal expression of TRAF6 in immune cells plays an important role in the antitumor response. The antitumor efficacy of Th9 is related to the upregulation of TRAF6 expression. TRAF6-/- Th9 cells have lower frequencies and decreased proliferation, and the insufficient persistence of TRAF6-/- Th9 cells also nullified their antitumor ability. This effect appears to apply only to Th9 cells because the antitumor function was similar between wild-type Th17 and TRAF6-/- Th17 cells in vivo [133]. In addition, TRAF6-deficient Tregs are dysfunctional in vivo. Mice with the Treg-restricted deletion of TRAF6 are resistant to implanted tumors and display enhanced antitumor immunity [134]. Similarly, the TRAF6 inhibitor decreases the population of intratumor Tregs by impeding the migration of Tregs towards the tumor, thus enhancing T cell-mediated antitumor immunity and thereby inhibiting the growth of Hepa1-6 tumor in immunocompetent mice, but not in immunodeficient mice [135]. In view of the dynamic interaction between cancer cells and the microenvironment, it is important to consider how the expression of TRAF6 in immune cells affects cancer progression.

The TRAF6 protein can be used as a drug target protein for cancer differentiation therapy. Through virtual screening, Chen et al. propose that the TCM compounds diiodotyrosine and saussureamine C may be potential candidate compounds as TRAF6 protein anticancer drugs [136]. A combined treatment of ionizing radiation (IR) with a proteasome inhibitor (MG132) shows synergistic cell-killing effects and induces endoplasmic reticulum stress in human pancreatic cancer cells, which results from the increased autophagy induction through the inhibition of TRAF6 [137]. In addition, TRAF6 has a carcinogenic role in the development of human oral cancer cells, and bortezomib and IR treatment can inhibit the growth of oral tumors. Bortezomib reduces the expression of TRAF6 protein through autophagy-mediated lysosomal degradation. Bortezomib also inhibits the IR-induced TRAF6 ubiquitination and TRAF6-mediated Akt activation. Therefore, the combination of proteasome inhibitors, IR therapy, and TRAF6 inhibition may be a new therapeutic strategy in oral squamous cell carcinoma [138]. Bortezomib induces apoptosis in myelodysplastic syndrome and acute myeloid leukemia through the autophagy-mediated degradation of the TRAF6 protein [139]. Rigosertib, a multikinase inhibitor, has selective cytotoxicity to diffuse large B cell lymphoma, which is related to its ability to reduce the expression of unmodified and sulfonated TRAF6 and inhibit the nuclear entry of TRAF6 [140]. Cinchonine, a member of the Cinchona alkaloid family, could complex with the RING domain of TRAF6, leading to the disruption of the binding with its natural ligand, the Ubc13 protein, thereby inhibiting the AKT and TAK1 signaling pathways and inducing the apoptosis of cancer cells [141]. Qian et al. switched the RING domain mutations of TRAF6 to the corresponding sequences in the rest of the TRAF family members, which all showed defects in their interaction with Ubc13, and confirmed that these substitutions could underlie the failure of these TRAFs to interact with Ubc13. The RING of TRAF6 does not function alone, but functions together with the neighboring sequences in TRAF6, including the residues preceding the RING, and the first zinc finger plays a structural role [142]. These findings indicate that the interaction of TRAF6 with Ubc13 is specific for TRAF6, but not for the other members of the TRAF family, highlighting the unique critical biological roles of TRAF6 in multiple signaling pathways. Therefore, the RING domain of TRAF6 can be regarded as a potential antitumor target. Doxorubicin is one of the most effective molecules used to treat various tumors. Interestingly, doxorubicin-mediated cell death is particularly dependent on TRAF6, and the TRAF6 binding peptide protects the doxorubicin-mediated late induction of NF-*κ*B and cell death [143]. In short, TRAF6 may represent a new cancer prognostic biomarker or potential therapeutic target.

## 7. Conclusion

In this review, we mainly discuss the role of TRAF6 in immune cells and its relationship to the development of certain autoimmune diseases and cancer. Although TRAF6 plays an indispensable role in regulating the development, homeostasis, and activation of immune cells, the excessive activation of immune cells may be a cause for the development of autoimmune diseases. How to balance the relationship between the activation of immune cells and autoimmune diseases is a problem that needs to be further explored. In fact, in addition to autoimmune diseases and cancer, TRAF6 is also involved in the pathogenesis of other diseases, including cardiovascular diseases [144] and central nervous system diseases [145]. Our progress in understanding the regulation of immune responses by TRAF6 will prompt us to develop new therapeutic strategies. Drugs can be developed to target the RING domain or TRAF domain of TRAF6 to modulate the activity of E3 ubiquitin ligase of TRAF6 and protein to protein interactions. In the future, we hope to have a more comprehensive understanding of TRAF6 and further facilitate the use of TRAF6 as an effective therapeutic target for multiple diseases.
